# On the topical issue of assisted dying: the insurmountable challenges of human existence, and the right to exit

**DOI:** 10.3389/fpubh.2026.1695897

**Published:** 2026-02-05

**Authors:** Tobore Onojighofia Tobore

**Affiliations:** Independent Researcher, Yardley, CA, United States

**Keywords:** assisted dying, assisted suicide, cruel dynamism of life, euthanasia, evolutionary imperfections and genetic predisposition to diseases, human nature, medically assisted dying, MAiD

## Abstract

Although the issue of medical-assisted dying or medical aid in dying (MAID, euthanasia, and medical-assisted suicide) has a long history, it has become an increasingly important topic in recent years. Around the world, many governments remain opposed to MAID. In places where MAID is legally permitted, it is highly regulated, and typically, unbearable suffering limited to a few serious and incurable medical conditions determines eligibility. MAID eligibility based on unbearable suffering but limited to a few medical conditions is unfair because suffering is inescapable in life, is not limited to medical conditions, and is highly subjective. Indeed, the subjectivity and individual differences in the experience of pain and suffering make it inaccurate to suggest that suffering is lesser for a person dealing with trigeminal neuralgia, extreme poverty, treatment-resistant depression, or the grief of losing a loved one compared to someone with a MAID-eligible condition. The subjectivity and individual differences in the experience of suffering mean that its intolerability can only be fairly defined by the individual experiencing it. So, the use of a third party (e.g., a physician) to evaluate severity, burdensomeness, or prognosis in determining MAID eligibility unfairly restricts people suffering who may not meet the clinical or physician’s threshold of intolerability. Also, suffering is often imposed on humans by forces outside their control, making denying the right to exit suffering with dignity through MAID unfair. In this position paper, an argument is made for MAID to be made broadly legal without age limits and available to anyone who requests it based on patient-defined unbearable suffering or fear of future suffering.

## Introduction

Euthanasia, the intentional causation of death by a physician, has a long history of controversy. Euthanasia can be voluntary, i.e., at the patient’s request; involuntary, in which the patient is put to death against their will; and non-voluntary, when the patient, because of decisional incapacity, is unable to consent or object to euthanasia (1). Medically or physician-assisted suicide (MAS) occurs when a physician plays a role in a patient’s death by supplying the means and/or information to assist the patient in performing the life-ending act. Both voluntary euthanasia and MAS are broadly categorized as medically assisted dying or medical aid in dying (MAID), and both involve a physician, with the difference being that in MAS, the patient takes the final action leading to their death (2, 3). The story of MAID as a good death is replete with passion from proponents and opponents because it generates conflicts at different levels, including moral, sociocultural, and ethical.

Opposition to MAID remains strong around the world, and there are several reasons for this situation. In ancient times, Hippocrates and his popular Hippocratic oath sought to protect patients’ lives through medicine, particularly in vulnerable health conditions with a high likelihood of fatal outcomes. The oath remains one of the foundational arguments used by those who oppose MAID (3, 4). Many physicians and medical associations remain uncomfortable with performing Euthanasia or MAS (5–9) in part due to the Hippocratic oath (10). In the Middle Ages, religion, particularly Catholicism, which was vehemently against suicide, influenced the thinking about euthanasia (3). During the Renaissance, a period of decline in the church’s authority and power and rise in humanism, helping the dying person achieve a dignified death without suffering was considered necessary (3, 4). In the 20th century, several important historical events caused the term euthanasia to stir up intense negative emotions. Sterilization of the rejected, including the blind, deaf, intellectually disabled, people with epilepsy, criminals, and rapists, was practiced in England, Sweden, and the United States (4). Nazi experiments in which the term euthanasia was essentially linked to murder, as opposed to a kind and compassionate act, contributed greatly to the negativity associated with euthanasia (11). During the Nazi Euthanasia program, about a quarter of a million subjects who had a physical or mental disability were killed (3). For Hitler and his regime, euthanasia represented the methodical elimination of those whose lives they considered undesirable and unworthy of living (3).

Today, the openness of modern societies, the increase in life expectancy, and chronic diseases that compromise the quality of life of people have sparked interest and discussion about MAID. Many countries worldwide remain vehemently opposed to MAID, with a few exceptions. In places where it is allowed, unbearable suffering (loss of autonomy, dignity, or intolerable pain) is the central criterion that governs such legislation and eligibility (12, 13). MAID policies vary wildly in places where they are permitted. Primarily, patients with a terminal condition such as cancer are eligible (2, 4). Although permitted in a few countries for patients with non-terminal illnesses, it remains highly controversial (2, 14). Some places permit it only for adults, while others have no age limit. In some places, euthanasia is not permitted, but MAS and some allow both. In Netherlands, MAID is permitted for adult patients (those aged 12 through 17 years require parental involvement) experiencing unbearable suffering with no chance of improvement. However, patients are not entitled to euthanasia, and doctors are under no absolute obligation to perform it (15). In Belgium, euthanasia is legal for people who experience unbearable suffering that cannot be relieved, and that is caused by a serious and incurable medical condition (14, 16). Eligibility is also extended to patients who are not terminally ill, such as those with psychiatric disorders (2, 14, 17). Belgium is the first country to legalize euthanasia for children without any age limit (18). In France, MAID is prohibited, but a bill allowing it strictly for French citizens or residents over the age of 18 with incurable conditions is currently undergoing discussions in the national assembly. In Switzerland, euthanasia is illegal, but it is one of the few countries in which MAS does not require that the patient be terminally ill. It can be done without the involvement of a physician and is not considered criminal if motivated by non-selfish considerations (19). Swiss law reduces the situations in which assisted suicide is a crime, and as a result, decriminalizes it in other cases (19). In Canada, MAID has been legal since 2016, but patients with mental illness as the sole underlying medical condition remain ineligible potentially till 2027. In the United States, euthanasia is illegal. However, several states have legalized MAS with restrictions, but many others prohibit it (15). In those states where MAS is approved, eligibility is limited to patients with terminal or irremediable medical illnesses and less than 6 months to live (20).

The Hippocratic oath and concerns about abuse because of historical reasons remain an issue with MAID. However, one key reason for the persistent opposition to MAID is religion. Research indicates that opposition to MAID is largely based on religious-based moral positions (21–23). The world’s most important religions in terms of the number of adherents (Christianity, Islam, Judaism, Buddhism, and Hinduism) are fundamentally against the moral acceptability of Euthanasia and MAS (24, 25). Euthanasia is prohibited in Islam, and many Christian denominations have expressed broad or specific opinions against suicide, MAS, and euthanasia, using biblical teachings that advocate that life is a sacred gift of God (25).

The denial of MAID to patients who request it to end their suffering is arguably the preeminent sociocultural, moral, and ethical issue of our time. It is fundamentally an issue of human dignity, autonomy, and above all, fairness. The objective of this paper is to discuss the unfairness of MAID prohibition or eligibility restrictions by discussing the inescapability and subjectivity of suffering, the fact that human suffering is not limited to medical conditions, and that most human suffering is imposed by forces outside their control. Also, to discuss the need to correct this unfairness by legalizing MAID as a global human right for all adults based on patient-defined unbearable suffering or fear of future intolerable suffering (the right to exit, or RTE).

## 1 Humans suffering due to forces outside their control

### 1.1 Evolution of subjective experience of pain/suffering

The evolution of feelings (negative affect and pain) is believed to contribute to fitness (26). However, pain and suffering are subjective and depend on individual differences in emotional, physiological, and cognitive states (27). This entails that the experience or perception, and interpretation of pain and suffering, is variable across individuals, and this is perfectly captured by the popular maxim by McCaffery (28) that “pain is what the person says it is and exists whenever he or she says it does.” The evolution of conscious experiences and the development of self-recognition and selfhood (29), which allows humans to reflect on their actions, the actions of others towards them, and the nature of things, means that humans are vulnerable to subjective suffering from unpleasant life events and existential issues. Furthermore, humans have evolved adaptations connected to conscious experience that impede happiness (30). These evolved adaptations, which include subjective distress emotions (e.g., psychological pain, anxiety, depression, anger, fear, shame, etc.) and competitive evolutionary adaptations (e.g., envy, jealousy) (30), are a major source of psychological suffering.

### 1.2 Notable evolutionary imperfections that impose medical suffering

Evolutionary imperfections, human genetic failures, and the mismatch between ancient evolutionary environment and modern environment/lifestyle predispose people to an array of serious medical conditions, many of which are outside human control, causing intolerable suffering. Some of these conditions have no effective treatment, are poorly understood, and the diagnostic journey can be long and challenging, causing significant suffering. For those with some effective treatment to manage them, the condition and, in many cases, the treatment impose significant physical, emotional, financial, and social burden on patients, leading to reduced quality of life.

#### 1.2.1 Rare diseases

Rare genetic diseases are believed to be the result of a constant forward genetic experiment that nature is conducting on humans (31). About 80 percent of rare diseases are due to genetic disorders (32). Globally, over 6 million people are affected by primary immunodeficiencies, which are caused by genetic defects that compromise some components of the immune system (33). Devastating neuromuscular diseases, which affect about 15 million people globally, are most often genetic (34). They result in significant incapacity and, in many cases, almost complete paralysis and ultimately death. Across the world, over 400 million (35) people suffer from about 7,000 different types of rare diseases, many of which are etiologically poorly understood, have no approved treatment options, and cause patients significant physical suffering, financial difficulties, and psychological despair (36, 37).

#### 1.2.2 Neurological conditions

Pain can be acute or chronic. Acute pain carries survival value to help inform the individual of tissue damage or a problem with body integrity. When acute pain persists beyond the healing process, it progresses to a chronic state, which can be considered a disease. Pain chronicity is a consequence of the evolutionary process (38). Evolutionarily, it is believed that human susceptibility to pathological pain, which is a maladaptive by-product of neural plasticity and pain mechanisms, may be because natural selection has evolved mechanisms that adaptively respond to repeated tissue damage by lowering the pain threshold and elevating pain salience (39). Many chronic pain conditions arise due to imperfect evolutionary adaptations or trade-offs. The evolution of bipedal locomotion in humans resulted in significant morphological adaptations of the skeleton, which, unfortunately, often lead to common physical pain conditions, such as hip fractures (40). Back pain is one of the greatest contributors to disability globally, with more than 50 % of people living in developed countries experiencing it at some point in their lives (41, 42). Research indicates that common sources of back pain, intervertebral disc herniation and spondylolysis, are linked to vertebral shape and the evolutionary shift from quadrupedalism to bipedalism (43, 44). Chronic pain is a major contributor to human suffering, associated with many diseases and conditions (45). It is linked with accelerated memory decline, a significantly increased risk of dementia (46), and early mortality (47). Furthermore, it negatively impacts multiple aspects of the sufferer’s health, including sexual function, mood/mental health, sleep, cardiovascular health, cognitive processes and brain function, and overall quality of life (48). In older adults, chronic pain leads to significant hardship, social isolation, and disability (49), and treatment with pharmaceuticals is typically only partially effective and limited by side effects (49). It affects more than 30 percent of the global population (50)^,^ and accounts for 15 to 20 percent of physician visits (51). By some estimates, the prevalence of chronic pain is almost 40 percent in developed countries and over 40 percent in developing countries (52).

Furthermore, one in two women and one in three men will develop neurological diseases such as dementia and Parkinsonism during their lifetime (53) and about 50 percent of the population may experience at least one episode of depression in the course of their life (54). This is in part because the brain is a highly complex organ, and its evolved complexity makes it vulnerable to trauma and dysfunction. Human evolution of higher cognitive function makes the brain highly susceptible to neurodevelopmental and neurodegenerative diseases (55). Research indicates that evolutionary alterations in human brain connectivity or circuitry in favor of more enriched and higher-order neural functionalities may have potentially made the brain highly vulnerable to dysfunction (56). Indeed, it has been hypothesized that human evolutionary development of language may have predisposed the brain to schizophrenia (57) and psychosis (58). Weak long-distance cortico-cortical connections in the more complex cortex may elevate susceptibility to disconnection syndromes like schizophrenia and Alzheimer’s (59). The increased capability of the human brain to engage in higher cognitive functions came with hefty metabolic and energy requirements, such that alterations to the brain energy utilization or impairment of energy metabolism increase susceptibility to cognitive decline and neurodegenerative diseases (60). Energy metabolism-related genes are involved in both the evolution and maintenance of human-specific cognitive abilities, and many of the genes and metabolite functions are disrupted in schizophrenia (61). Posttraumatic stress disorder, a source of suffering for many people after a traumatic event, is hypothesized to be the byproduct of imperfect brain evolutionary processes (62).

Also, the evolution of the human brain makes it vulnerable to drug and behavioral addictions. Drug and alcohol addiction have been a serious public health concern throughout human history and are major contributors to global disease burden and disability (63). Addiction occurs because humans evolved behavior regulation mechanisms that are rooted in neurochemical transmitters (64). Indeed, human behavior is regulated by ancient systems of serotonergic and dopaminergic networks (65), and 5-HT (serotonin) and cortico-mesolimbic dopaminergic systems are the target of many drugs. The motivation to relentlessly pursue and consume rewards has evolutionarily been driven by the urge to satisfy physiological needs and improve fitness. Psychoactive substances exploit the circuits that are normally triggered by an event that provides a gain in fitness, but they provide no fitness (64). Substance abuse may also be due to the brain’s evolved need for pleasure/rewards, as people who have fewer sources of pleasure in their daily lives are more vulnerable to substance abuse (64).

#### 1.2.3 Autoimmune diseases

Autoimmune diseases are very diverse and include over 70 different disorders encompassing endocrinological, rheumatic, neurological, and gastrointestinal diseases. They are marked by dysregulation of the immune system following exposure to proinflammatory environmental agents, resulting in clinically apparent pathology. Autoimmunity and autoimmune diseases are significantly increasing in many places globally (66) and represent a significant cause of illness and morbidity. Autoimmune diseases are largely because humans have evolved an immune system that is vulnerable to molecular mimicry, which is one of the critical means by which infectious or chemical agents may cause autoimmunity (67, 68). Molecular mimicry occurs because pathogens express or mimic proteins that resemble host proteins, and this provokes immune responses that are cross-reactive to both the mimicked and mimicking proteins (67). While mimicry may help to induce immune tolerance, it is often a harmful stimulus for the immune system due to its contribution to autoimmunity (67, 68). Indeed, chronic autoimmune diseases are a byproduct of the immune system misrecognizing self-antigens as foreign (69). Significant indirect evidence suggests that molecular mimicry contributes to the loss of self-tolerance in various autoimmune conditions (70).

Infectious pathogens played a critical role as selective forces that shaped the human genome and evolution (71), and genetic studies suggest the role of pathogen-driven selection pressures in the increased prevalence of autoimmune diseases (72–74). The genetic foundation of common autoimmune diseases may, in part, be explained by positive selection events, which both increased fitness and susceptibility to disease (75). Indeed, genetic variants that played a critical role in protecting people from the plague pandemic (Black Death), caused by the bacterium *Yersinia pestis*, overlap with alleles currently linked to increased susceptibility to autoimmune diseases (76). Several lines of evidence indicate that in Sub-Saharan Africans, specific genetic variants in the Apolipoprotein L1 gene (G1/G2 risk alleles), believed to provide immunity against African human trypanosomiasis, significantly increase susceptibility to severe lupus nephritis and progression to end-stage renal disease (77, 78). Ancestral human leukocyte antigen class II haplotypes selected in an environment of greater infectious load and preserved because of their ability to provoke powerful T-cell reaction against pathogens may be linked with a higher tendency toward immune hyperreactivity and autoimmunity in modern environments (79).

The human gut harbors and evolved with trillions of diverse microbes collectively known as microbiota or microbiome, which play a crucial role in wellbeing (80). The gut microbiota can be easily disturbed (81), and its functional and compositional alteration may cause the immune system to be wrongfully directed in favor of pro-inflammatory pathways, instigating different autoimmune processes (82). Evidence indicates that a translocating gut pathobiont, *Enterococcus gallinarum,* can provoke pathological human adaptive immune responses, specifically B-cell and T-cell dependent autoimmune responses (83). Aging is a significant risk factor for autoimmune disease, and research suggests that immune aging results in damage and failure in fundamental processes in immune cells, including mitochondrial dysfunction, lysosomal failure, and endoplasmic reticula stress, which induces macrophages and pathogenic T cells that promote autoimmune diseases (84). Many autoimmune diseases occur more frequently in women, and some research suggests that this is a genetic predisposition due to their second X chromosome (85, 86). Also, the mismatch between ancient evolutionary environments and modern societies/lifestyles of reduced immune challenges, increased lifetime ovarian hormone exposure, and cyclical immunomodulation has been hypothesized to play a role in the sex disparity and rise in autoimmune disease prevalence (87).

#### 1.2.4 Cancers

Cancer is the first or second most common contributor to early death around the world, and among all human diseases, it imposes the greatest social, clinical, and economic burden with regard to cause-specific Disability-Adjusted Life Years (88, 89). Cancers are caused by mutations that may be genetic, triggered by environmental factors, or due to DNA replication errors (90). Due to humans’ relatively large body size and long lifespan, the evolutionary process required the development of capable and potent tumor-inhibiting mechanisms to limit the development of cancers (91). However, this system is weakened or overwhelmed at post-reproductive age and by modern lifestyle and environmental changes (chemicals, pollutants, to radiation). These carcinogens may promote both the pathogenesis of cancer and its evolution by increasing the frequency of mutations and selection for adaptive mutations (91). All tissues are not equal in the emergence of cancers, and the lifetime risk of many types of cancers is significantly linked with normal self-renewing cells, total number of cell divisions. Indeed, the risk of most cancers is due to random bad luck or random mutations that occur during DNA replication in healthy stem cells (90, 92). In other words, regardless of lifestyle, environment, and genetics, the risk of cancer exists for all humans due to the evolutionary imperfection inherent in cell division. The estimated lifetime risk of cancer from birth to death is about 25 percent or one in four persons developing it (93).

The mismatch between the ancient evolutionary environment and modern society and lifestyle also contributes to the rise in the incidence of cancer. Indeed, younger people are being diagnosed with cancer more than ever, and Western-style diet, obesity, sedentary lifestyle, microplastics, and antibiotic use, particularly in early prenatal to adolescent periods of life, have been implicated in the rising risk of cancer (94–96).

#### 1.2.5 Cardiovascular diseases

The cardiovascular system is highly vulnerable to disease. Cardiovascular events (heart attacks and strokes due to atherosclerosis) are the predominant cause of deaths globally, and evidence suggests that human evolutionary loss of cytidine monophosphate-N-acetylneuraminic acid hydroxylase (CMAH) expression may promote a predisposition to atherosclerosis via different intrinsic and extrinsic mechanisms (97). Human ancestral environment had a greater infectious load, leading to a positive selection for pro-inflammatory genes and a parsimonious genotype, which caused fat accumulation, and the presence of these ancestral genes and mismatch with the modern environment and lifestyle increases susceptibility to metabolic and cardiovascular diseases (98). The cerebral vascular system is highly vulnerable to the formation of aneurysms, which are associated with negative outcomes on rupture. Blood vessels are vulnerable to dysfunction and injury due to multiple factors, including infections, changes in hemodynamic factors, cell proliferation, inflammation, aging, and failures in the blood clotting system. Lifetime risk estimates for total cardiovascular disease are greater than 30 percent for all individuals. For men, it is 60 percent at 45 years of age, and for women, it is about 56 percent (99).

### 1.3 Human suffering is not limited to disease/medical conditions

Several features of human existence impose suffering that may not result in a medical condition, as they are just part of the nature of things. Although there is counselling, different forms of support, and treatments, they impose significant suffering on people.

#### 1.3.1 The cruel dynamism of life events

Life is a dynamic place, and things always tend to change. This dynamism, often beyond the control of people, brings a great deal of pain and suffering. We live our lives at the mercy of the vagaries of this dynamism. People lose their jobs, and the stock market and investment value may crash unexpectedly, leading to financial ruin. The illusion of stability can be shattered at any time. Unforeseen man-made catastrophes and natural disasters can occur, such as tsunamis, earthquakes, tornadoes, wildfires, flooding, etc., destroying lives and properties and causing significant suffering. Shocking tragedies can occur at any time, changing life fortunes and causing enduring pain. Imagine the pain of the loved ones of the passengers of Malaysia Airlines MH370 that went missing in March 2014. A routine flight suddenly turned into a nightmare for many people. A significant tragedy can swing a person’s life suddenly from relative happiness to an existential crisis. This cruel dynamism of life means that tragedy is never far away, no matter how well a person behaves or plans, and this is a critical feature of human existence. Traumatic life events are associated with a high risk of suicidality (100–102), indicating that they are unbearable for many people.

#### 1.3.2 The cruelty of human nature

There are proponents of the idea that people are fundamentally bad, and there are those who argue that people are fundamentally good, altruistic, compassionate, and capable of recognizing right from wrong. Hobbes and Rousseau proposed opposite theories of human nature and morality. Hobbes’ view of humans was low, and he believed that humans were naturally selfish. Like Hobbes, Niccolò Machiavelli in *The Prince* described humans as untrustworthy, dishonest, timid, and greedy (103, 104). In contrast, Rousseau believed that all people are born good, but corrupted by civilization, and evidence supports the view that people may be inherently good and altruistic, and this altruistic behavior may be unique in the animal world (105).

The truth about human nature lies somewhere in the middle. Both arguments are flawed because they view good and bad as mutually exclusive behaviors. Humans are much more complex than such simple terms can illustrate. People are neither fundamentally good nor bad; they are both. Humans could be placed on a spectrum with good on the right and bad on the left. A person’s position on this spectrum is fluid and influenced by many factors. Although all people fall somewhere on that good/bad spectrum, most people often linger on the good side with a high potential to be on the bad side. Driven by the basic evolutionary need to survive and thrive, people can be cruel to each other, and a great deal of the suffering experienced in life is imposed by fellow humans. Indeed, the average human, while capable of altruism, is susceptible to vanity, narcissism, selfishness, and hate, can be easily corrupted by power (106), and aroused to envy, can engage in bullying, revenge, negative gossiping, and *schadenfreude* because of their competitive nature (30), and, in pursuit of their perceived interests, are potential hypocrites, cheats, thieves, and killers.

Human violent, aggressive nature (107–109) means they are willing to cause both physical and psychological harm to others in furtherance of their goals. About 1 in 3 women worldwide have been subjected to some form of violence in their lifetime (110). Worldwide, millions of children, men, and women suffer from interpersonal violence, including youth violence, child maltreatment, intimate partner abuse or aggression, abuse of older people, and sexual violence (111). Research indicates that about 40 million people worldwide are victims of human trafficking (112). In 2021, 50 million people were reported to be living in modern slavery (113). The wars for territorial conquest, hegemony, crimes against fellow humans, and the development of weapons of mass destruction provide great insight into humans’ violent nature. In 2023, there were almost 200 ongoing regional and local conflicts globally (114), displacing millions of people as refugees. Combined with climate change and economic inflation, 2023 was a year of significant humanitarian catastrophes. Notably, the challenges of 2023 are not the exception but the norm of much of human existence. Indeed, human history of the last few centuries is noted by mass murders and conflicts (115). Wars triggered by the political elites impose significant suffering on ordinary people who had nothing to do with the events that triggered it, but must live with the consequences. Many face displacement, loss of income and property, untimely death of loved ones, violence, injury, health issues, etc.

Human deceit is a source of suffering, with many people and organizations being victims of different scams that strip them of their life savings and identity (116). Prejudice is a component of human nature (117, 118), and all forms of prejudice, bias, discrimination, and hate make life incredibly difficult for those at the receiving end of such acts. Prejudice from racism, sexism, ageism, religious affiliation, tribalism, casteism, disability, classism, gender identity, culture, nationalism, etc., interferes with the happiness and quality of life of people on the receiving end of such acts. Indeed, discrimination and racial prejudice are linked with adverse health outcomes, lower subjective cognitive function, poor mental health, well-being, and substance use (119–124). The cruelty caused by fellow humans is a major source of suffering in life.

## 2 Discussion

Previous arguments on MAID legalization have often focused on patient autonomy, dignity, compassion, empathy for people with terminal and incurable diseases, and the financial burden of chronic diseases on families. Also, discussions on MAID have been mostly limited to medical conditions, especially terminal conditions, and MAID for patients with non-terminal illnesses remains controversial (2, 14). This article takes a different approach by emphasizing the fact that suffering is an inescapable aspect of human life, largely imposed by forces outside human control, including evolutionary and genetic imperfections and the mismatch between ancient evolutionary environment and modern society/lifestyle that predispose people to a vast array of diseases/conditions. Also, highlighted is the subjectivity of suffering, and the fact that suffering is not limited to disease/medical conditions due to human cruel nature and the cruel dynamism of life events.

The subjectivity and individual differences in the experience of suffering mean that its intolerability can only be fairly defined by the individual experiencing it. Consequently, the use of a third party (e.g., a physician) to evaluate severity, burdensomeness, or prognosis to determine MAID eligibility unfairly restricts people suffering who may not meet the clinical or physician’s threshold of intolerability. Furthermore, the fact that suffering is largely imposed on humans by forces outside their control means that preventing people from escaping their suffering with dignity through MAID if that is their choice is unfair. The prevention of a person suffering from complex regional pain syndrome from MAID is not just unfair because it is a condition imposed by evolutionary imperfections; it is tantamount to a miscarriage of justice and double punishment, which is illegal in most countries. There is no justification for prohibiting or excluding a person interested in MAID who is dealing with severe drug addiction with an endless cycle of recovery and relapse, and who has lost everything in their life due to the addiction. Imagine the case of Diane Pretty, who is suffering from motor neuron disease and is experiencing progressive disintegration of her body, but was denied her request to end her suffering through MAS (125). She is experiencing incalculable suffering imposed on her by no fault of hers. Similarly, the case of two severely disabled victims of locked-in syndrome, who wished to end their immense suffering, through MAID, but were denied (126). From all indications, they were living in intolerable suffering but were denied the right to end their suffering. There are also cases of patients who are suffering from serious illness attempting suicide after being unjustifiably (taking into account the subjectivity of pain and suffering) found ineligible for MAID (127). Justice based on fairness and quality is at the heart of the world we all aspire to create and live in, and the unfair restriction or prohibition of people from escaping intolerable suffering defeats this goal.

Taking into account that suffering is subjective, inescapable, not limited to medical conditions, and imposed on humans largely through no fault of their own, MAID prohibition and restrictions should be lifted, decriminalized, and legally provided based on patient-defined unbearable suffering (the right to exit, or RTE). This unbearable suffering is defined solely by the patient and does not require any third-party (e.g., physician) assessment of the severity, burdensomeness, and prognosis of the patient’s suffering. Thus, it could be due to any reason that is causing a patient suffering, such as existential suffering (128), extreme poverty, terminal or nonterminal medical conditions, traumatic events, etc. The fear of future unbearable suffering for different reasons should also be eligible, including in the early stages of an incurable disease (such as a neurodegenerative disease, or cancer), diagnosis of a chronic disease that negatively impacts the patient’s quality of life for which they do not wish to undergo treatment, etc. RTE should have no age limits. However, it should remain limited to terminal, incurable medical conditions for children under 18 with parental involvement/approval. The patient/applicant must directly make the request and provide a reason for their unbearable suffering with their request. They must be counselled, informed of all available treatment options, and given all available support (social, financial, etc.) to improve their condition. People with pending criminal or civil cases or those serving a sentence for a conviction should be excluded while incarcerated to ensure that criminal/legal liability is not evaded. However, people incarcerated with a terminal condition should be eligible.

Some will argue that humans are not entirely blameless, as lifestyle choices that people independently make play a role in suffering from diseases. While there may be some truth to this argument, many lifestyle choices that people make are influenced by genetics (e.g., personality), the environment they were born into, and evolutionary imperfections (vulnerability to addictions) (64). Even with healthy lifestyle choices, people remain vulnerable to diseases due to evolutionary imperfections and the mismatch between ancient and modern environment/lifestyle.

Some may argue that humans have made enormous progress in creating a better world, and instead of legalizing MAID, efforts should be geared toward reducing suffering. Human ingenuity has indeed resulted in significant achievements, making life better than it has ever been. Humans have achieved so much in the last 250 years in almost every sphere, including medicine, telecommunications, science and technology, transportation, etc. However, even with our best efforts, life will remain an incredibly challenging experience. Even with our best efforts, human life can never be free of disease due to the physiological complexity of the human body, the multi-etiological factors that impact many diseases, the significant number of evolutionary imperfections in the human body, the discrepancies between ancient and modern environments, the significant number of pathogens, etc. Due to flawed human nature, there will always be people struggling to fit in and facing significant prejudice and barriers as they navigate life. Poverty is unlikely to be eradicated because of government resource constraints, lack of political will, and the innumerable disparities and inequalities between people. Even in rich countries where poverty is low, and life is comparatively better, the incidence of suicide and suicide attempts is fairly high (129), suggesting that the triggers of human suffering are complex and innumerable. For the few who are socioeconomically successful, money and power may ameliorate some of the sufferings of existence, but do not provide immunity against the abundance of disease, cruelty from fellow humans, the cruel vagaries of life, and the evolved adaptations that interfere with happiness (30).

Some will argue that allowing MAID for conditions for which a patient can get some relief from treatment is wrong. Indeed, the idea that the patient’s suffering should be unrelievable or that there is no chance of improvement from treatment is one of the eligibility criteria of many MAID laws. Similarly, some have argued that palliative care has significantly improved and should be adequate to counteract the chronically ill’s need for MAID (15, 130, 131). Both arguments fail to consider the subjectivity and individual differences in suffering and the fact that most diseases/conditions are a consequence of genetic and evolutionary imperfections imposed on patients. These arguments interfere with patients’ freedom to reject treatment in favor of dying. Treatment, which, while in many cases is beneficial due to advances in modern medicine, is burdensome and may likely come with significant side effects.

Also, arguments may arise about the potential for coercion if MAID is broadly legalized. Indeed, opponents of euthanasia have argued that legalizing it will result in it being used without a patient’s explicit request, particularly in vulnerable patient groups. However, all available evidence from studies on MAID before and after legalization in Oregon and the Netherlands invalidates this concern (132). With the few nations that have permitted MAID, there is no evidence of abuse such as suicide contagion, an increase in non-voluntary forms of life-ending, or expansion to minors (4, 133). However, vigilance towards coercion, consent, and capacity must remain. Concerns have been raised about physicians’ use of continuous deep sedation and medico-legal imperatives like the Principle of Double Effect to hasten patients’ death (134, 135), and more efforts and clarity should be put in place to avoid this. The broad legalization of MAID based on patient-defined unbearable suffering will stop the need for physicians to hide behind medical terminologies to fulfill the wishes of patients who are dealing with intolerable suffering.

Some will argue that allowing MAID in any capacity will send the wrong social message that it is an option for people to consider. This argument is flawed. Interest in living is deeply embedded in all living things, including humans, driven by deep evolutionary and sociocultural forces. MAID’s broad legalization based on patient-defined unbearable suffering will not suddenly send a signal to everyone to explore MAID. Indeed, there is no evidence of a spike in MAID requests in the 100 s of thousands or millions in places where it is permitted (4, 133).

Some may argue that suicide is something we strive to prevent as a society because of its lasting negative impact on families and communities (136). Why then should MAID be permitted? The truth is that MAID is different from suicide (137) (Table 1). Suicide and suicide survivorship are associated with stigma. It is a taboo in almost all cultures, unlike MAID, which is associated with compassion, autonomy, and dignity (20, 138, 139). Unlike MAID, suicide may be associated with impulsiveness (140), and mental illness is a significant risk factor for suicide (141), suggesting it may not necessarily be a rational choice. MAID follows a strict and highly regulated process where capacity, consent, and moral and legal justification are essential, and there is exhaustive reflection and contemplation on the patient’s part (137). Notably, most people who attempt or successfully commit suicide do not necessarily want to die (142) but just want to escape the stressful event that is disturbing them, which they feel helpless to overcome (143). In contrast, people who request MAID and remain so throughout the process are interested in ending their lives. This is why suicide should be prevented, and help should be given to people with suicidal ideation or feeling helpless in dealing with a stressful event.

**Table 1:** Differences between suicide and medically assisted dying.

Medically assisted dying (medically assisted suicide and euthanasia)	Suicide
The physician is involved; in euthanasia, the physician performs the act, while in MAS, the physician provides the drug.	The individual acts alone and, typically, in isolation to avoid being stopped.
Involves a highly regulated process, including an interview, discussion, and assessment, before being granted	Typically, individualized and may not necessarily be planned
Done in a safe environment with a life-ending drug; hospital/clinic, or at the patient’s home.	It could be done anywhere, including in public, using gruesome methods, and could create a public hazard.
People who request MAID wish to end their lives.	People who commit or attempt suicide do not necessarily wish to end their lives. They typically feel overwhelmed or helpless against their stress or pain.
Done after a highly regulated process, giving the patient time to think and reflect on their decision	It could be irrational and done out of impulse.
An extreme measure that is likely to be successful in ending the patient’s life	An extreme measure that may fail at first, leading to indelible physical, emotional, and sociocultural harm or negative consequences
Associated with patient autonomy, human rights, and dignity	It is a cultural taboo, associated with weakness and stereotyped as a mental illness.
Patients could close their affairs, and due to the legal nature of MAID, inform loved ones of their plans.	May not necessarily close their affairs and are unlikely to inform anyone so as not to be prevented or reported to relevant authorities.

Also, arguments have been made that MAID should not be permitted at all because life is valuable and all intentional killing is fundamentally wrong (144). This argument is flawed. Although society claims to value people with disabilities and would not permit MAID, many people with disabilities face significant barriers and discrimination in their lives from public policy, social services, and healthcare delivery, making it unendurable (145). Life may be valuable, but society tolerates people living dangerously or engaging in actions that promote their likelihood of early mortality, such as violent sports, eating unhealthy foods, cigarette smoking, and alcohol use. Individual freedom to choose is one of the arguments for why such things are permitted. Why then is that standard not applied to MAID for the individual to decide about their continued living based on their experience of unbearable suffering imposed on them, typically by forces outside their control? It is not illegal for people to live on the margins (extreme socioeconomic disadvantage and homelessness), which is associated with early mortality. Why should it be illegal for someone with unbearable suffering due to living on the margins to access MAID and end their misery with dignity?

Many physicians and medical associations remain uncomfortable with performing Euthanasia or MAS (5–9). The Hippocratic oath remains a critical rallying point for the argument against MAID. This interpretation of the Hippocratic oath is flawed because MAID is about relieving patients of suffering, which is in line with the oath. MAID is in line with patients’ interests and constitutes a moral good (125). Arguments have been made by the American College of Physicians (ACP) that the legalization of MAS may negatively impact trust in patient-physician relations and alter the medical profession’s role in society (8). There is no evidence in the published literature from places where MAID, MAS, or voluntary euthanasia is legal that the patient-physician relationship has been negatively impacted or that there have been changes in the public’s perception of the medical profession. The vehement rejection of MAID by medical associations is not rational and unnecessarily constrains the liberty of patients (10). It may compel some physicians to covertly and illegally engage in euthanasia by hiding behind medico-legal terminologies (134, 135). Shared decision-making is a critical part of clinical care, and to vehemently oppose or reject MAID is to ignore patients’ critical need to end their suffering. The Swiss approach to allowing non-physicians to perform MAS, as far as it is driven by non-selfish reasons, may be concerning for many people. This is why physician participation in the euthanasia or MAS process is critical to evaluate and confirm the patient’s cognitive status and consent, safeguarding the process from abuse. Medical training must endeavor to promote the importance of respecting patients’ MAID requests due to their experience of intolerable suffering, so that physicians may overcome the emotional burden and perceived sense of guilt that prevents their participation in MAID. Physicians have a positive duty based on the ethical principles of nonmaleficence, beneficence, justice, and autonomy (146), and these principles should be fundamental considerations in the provision of MAID. However, respectfully taking into account that some physicians may have strongly held personal beliefs that inform their views on MAID, and there are religious or spiritual, ethical, and public policy arguments about MAID, a physician who does not wish to perform MAID should have the right to refuse to provide it. If they wish, they should be able to transfer the patient request and medical file to a physician who is willing to do it.

Despite some negative history associated with MAID, the biggest obstacle to its legalization is religion. While religious belief should be respected, it should not be allowed to dictate policy on something as fundamental as the right to exit suffering as defined by the individual. The religious argument that life is always sacred and a divine gift and, therefore, cannot be interrupted sees suffering as part of God’s grand plan that should not be interfered with. This is not based on any evidence, and such a perspective is increasingly not shared by many people in society, as evidenced by the growing number of non-religious people in society. Most public policies are based on evidence, and the issue of MAID should be no different. The subjectivity and intolerability of suffering are real and not part of any grand divine or cosmic plan. The right approach is a secular and compassionate approach based on the individual’s suffering as defined by them and their wish to end their life.

In summary, current MAID policies around the world need to change or expand. Many countries’ MAID policies are totally against MAID for historical and religious reasons. For the few that permit it, although based on unbearable suffering, they typically unfairly restrict eligibility to a few terminal and incurable medical conditions. Suffering is an inescapable part of human existence, largely imposed by forces outside human control. Suffering is not limited to medical conditions, and it is highly subjective, meaning its intolerability is best defined by the individual. In furtherance of our efforts to build a fairer world, MAID should be legalized and made readily available as an option to everyone based on patient-defined unbearable suffering or fear of future suffering without any third-party (e.g., physician) assessment of severity, burdensomeness, or prognosis. This will create a fair standard for MAID as opposed to the current fragmented system, where every nation defines unbearable suffering and MAID eligibility using different criteria. RTE should be enshrined in all United Nations human rights charters and treaties, and the constitutions of all nations. Just as life is sacred, the right of one to choose to die with dignity by MAID is equally sacred and must be guaranteed. The world will be a better place for it.

## Data Availability

The original contributions presented in the study are included in the article/supplementary material, further inquiries can be directed to the corresponding author.
